# An immune exhaustion signature predicts prognosis and identifies patients with diffuse large B-cell lymphoma (DLBCL) who derive preferential benefit from chimeric antigen receptor (CAR)-T cell therapy

**DOI:** 10.1016/j.cpt.2026.02.002

**Published:** 2026-02-13

**Authors:** Bo Zheng, Wanqian Hu, Ying Fang, Rong Li

**Affiliations:** Department of Hematology, Navy Medical Center of PLA, Naval Medical University, Shanghai 200052, China

**Keywords:** Large B-cell lymphoma, immune exhaustion, immunotherapy, Biomarkers, Prognosis

## Abstract

**Background:**

The tumor microenvironment (TME) is a key determinant of prognosis in diffuse large B-cell lymphoma (DLBCL). While T-cell exhaustion is implicated in therapeutic failure, its precise molecular hallmarks and utility for predicting response to modern immunotherapies, such as chimeric antigen receptor (CAR)-T cell therapy, remain unclear.

**Methods:**

We performed an integrative analysis of transcriptomic and clinical data from multiple DLBCL cohorts (The Cancer Genome Atlas [TCGA], GSE181063, GSE10846, GSE248835, GSE182434). We used unsupervised clustering, exploratory analysis of single-cell RNA sequencing data, and the least absolute shrinkage and selection operator for variable selection (LASSO-Cox) regression to characterize the exhausted TME, construct a prognostic model, and evaluate its predictive value for CAR-T cell therapy. The model's dynamic behavior was assessed in a proof-of-concept longitudinal cohort of patients treated with the T-cell-engaging bispecific antibody glofitamab.

**Results:**

We identified a “high-exhaustion” subtype associated with significantly poorer overall survival (OS; log-rank *P* = 0.016). Based on this, we developed a five-gene immune exhaustion-Related Prognostic Score (IERPS) that served as a robust independent predictor of poor OS across multiple cohorts. Critically, in a cohort of 256 relapsed/refractory patients, the IERPS was strongly prognostic for event-free survival (EFS) in the standard-of-care (SOC) arm (HR = 2.02, 95% confidence interval [95% CI]: 1.07–3.81, *P* = 0.029) but lost prognostic significance in the CAR-T arm (HR = 0.70, 95 % CI: 0.35–1.40, *P* = 0.314). This significant interaction suggests that CAR-T cell therapy may abrogate the poor prognosis associated with a high IERPS. Biologically, exploratory single-cell analysis (*n* = 4 samples) defined the high-IERPS state by hallmarks of classical T-cell exhaustion, and a descriptive case study showed the score dynamically tracked clinical response to glofitamab.

**Conclusions:**

A state of active T-cell exhaustion and a suppressive TME drive the adverse immune phenotype in DLBCL. Our IERPS model captures this dysfunctional state, acting as a powerful prognostic tool and, more importantly, as a potential predictive biomarker to identify high-risk patients who appear to overcome their inherently poor prognosis through CAR-T cell therapy.

## highlights

• Single-cell analysis defines the adverse diffuse large B-cell lymphoma tumor microenvironment (DLBCL TME) as a state of classical T-cell exhaustion rather than immune quiescence.
• A robust five-gene score (immune exhaustion-Related Prognostic Score, IERPS) independently predicts poor survival across multiple cohorts.
• IERPS exhibits clinical significance in individualized medicine for chimeric antigen receptor (CAR)-T cell therapy.
• IERPS dynamically tracks clinical response to the T-cell engager glofitamab.

## Graphical abstract

**Figure:**
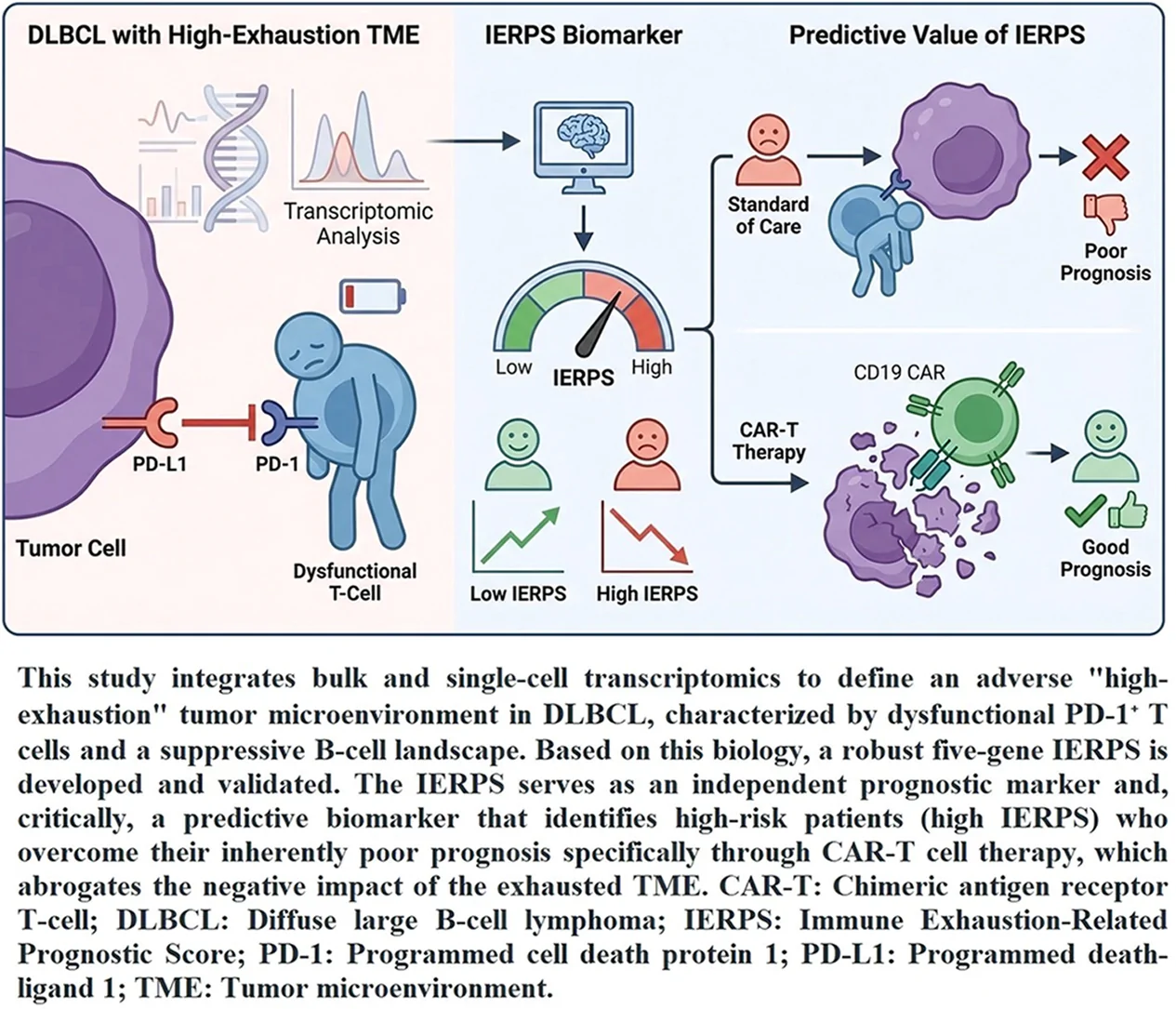

## Introduction

Diffuse large B-cell lymphoma (DLBCL) is the most common non-Hodgkin lymphoma, characterized by significant clinical and molecular heterogeneity.^1^^,^^2^ Despite advances in chemoimmunotherapy with rituximab, cyclophosphamide, doxorubicin also known as hydroxydaunorubicin, vincristine (Oncovin), and prednisone (R-CHOP), a substantial proportion of patients experience relapsed/refractory (R/R) disease, highlighting the urgent need for robust biomarkers to improve risk stratification and guide increasingly complex therapeutic decisions.^3^^,^^4^

The tumor microenvironment (TME) has emerged as a critical modulator of lymphoma progression and therapeutic response.^5^ T-cell exhaustion, a state of cellular dysfunction characterized by progressive loss of effector function and sustained expression of inhibitory receptors, is a key mechanism of immune evasion across various cancers.^6^^,^^7^ While a “T-cell-inflamed” yet exhausted TME has been linked to poor outcomes in DLBCL,^8^ its precise cellular and functional definition, particularly in the context of novel immunotherapies, requires deeper investigation.

The advent of chimeric antigen receptor (CAR)-T cell therapy, particularly targeting CD19, has revolutionized the treatment of R/R DLBCL.^9^^,^^10^ However, identifying which patients will benefit most from this intensive therapy is a major clinical challenge.^11^ A predictive biomarker should not only identify high-risk patients but ideally identify patients in whom a specific therapy may mitigate this risk. Similarly, T-cell-engaging bispecific antibodies like glofitamab represent another powerful therapeutic class, creating a need for dynamic biomarkers to monitor patient response.^12^^,^^13^

In this study, we hypothesized that a deep molecular characterization of the immune landscape could yield such a biomarker. We integrated bulk and single-cell transcriptomics to define the “high-exhaustion” phenotype, revealing it to be a state of classical T-cell exhaustion within a highly suppressive TME. We then developed and validated a five-gene prognostic score, the immune exhaustion-Related Prognostic Score (IERPS), based on this biology and tested its ability to predict which patients derive disproportionate benefit from CAR-T cell therapy, thereby addressing a critical gap in personalized medicine for DLBCL.

## Materials and methods

### Patient cohorts and data acquisition

This study utilized multiple publicly available datasets and one in-house cohort.

#### TCGA-DLBCL cohort

Normalized bulk RNA-sequencing (RNA seq) data (Fragments Per Kilobase of transcript per Million mapped reads [FPKM]) and corresponding clinical information for 481 DLBCL patients were downloaded from National Cancer Institute Center for Cancer Research (NCICCR)-DLBCL project via the Genomic Data Commons (GDC) Data Portal. This cohort was used for the initial discovery of immune exhaustion subtypes.

#### Model discovery and validation cohorts

i. **Discovery Cohort (GSE181063):** This dataset, obtained from the Gene Expression Omnibus (GEO) database, contains bulk RNA sequencing (RNA-seq) data and clinical follow-up for 1144 DLBCL patients. It was used as the primary cohort for the development of the prognostic model.
ii. **External Validation Cohort (GSE10846):** This microarray dataset (Affymetrix Human Genome U133 Plus 2.0 Array) with data from 420 DLBCL patients was downloaded from GEO and served as an independent external validation set for the prognostic model.

#### CAR-T cell therapy cohort (GSE248835)

This GEO dataset includes bulk RNA-seq and clinical outcome data for 256 R/R DLBCL patients treated with either standard-of-care (SOC) or axicabtagene ciloleucel CAR-T therapy. The RNA-seq was performed on tumor biopsy samples collected prior to the initiation of study therapy (either SOC or pre-lymphodepletion for CAR-T). It was used to evaluate the predictive value of the model.

#### Single-cell RNA-sequencing (scRNA-seq) cohort (GSE182434)

Pre-processed scRNA-seq data from four DLBCL tumor biopsies (two classified as “high-exhaustion” and two as “immune-exhaustion” based on TME profiles) were downloaded from GEO. This dataset, comprising over 10,000 cells, was used for in-depth TME characterization.

#### In-house longitudinal cohort

Peripheral blood samples were collected from two R/R DLBCL patients treated with Columvi (glofitamab) at our institution under an approved protocol. Samples were collected at three timepoints: pre-treatment (Pre), during-treatment (During), and post-treatment (Post). Bulk RNA seq was performed on these samples to assess the dynamic changes of the prognostic signature.

### Bulk RNA-sequencing data processing and analysis

For RNA-seq datasets (TCGA, GSE181063, GSE248835), gene expression levels were converted to Transcripts Per Million (TPM) and subsequently log2-transformed (log2[TPM+1]) for analysis. For the GSE10846 microarray dataset, probe-level data were processed, background-corrected, and normalized using the Robust Multi-array Average (RMA) algorithm, as implemented in the affy and limma R/Bioconductor packages. Probes were mapped to gene symbols, and for genes with multiple probes, the one with the highest average expression was retained.

Since the IERPS model was trained and validated on separate, unmerged cohorts, direct batch correction was not applied. The model's robustness across different datasets and platforms serves as a validation of its generalizability.

### Unsupervised clustering and subtype identification

In the TCGA-DLBCL cohort, unsupervised hierarchical clustering was performed based on the Euclidean distance and Ward's D2 linkage method using the expression of 15 well-established immune exhaustion-related genes. These genes were selected based on an extensive literature review to represent a comprehensive panel of canonical co-inhibitory receptors (e.g., programmed cell death 1 [*PDCD1*], cytotoxic T-lymphocyte associated protein 4 [*CTLA4*], T cell immunoreceptor with Ig and ITIM domains [*TIGIT*], lymphocyte activation gene 3 [*LAG3*], hepatitis A virus cellular receptor 2 [*HAVCR2*]), their ligands, and other key markers whose sustained co-expression is a hallmark of the exhausted T-cell phenotype as defined in seminal works in the field.^6^^,^^7^ This analysis was used to stratify patients into high- and low-exhaustion subtypes.

### Data and code availability

The datasets used in this study are publicly available from TCGA and GEO. All analysis code used to generate the results and figures will be made available on the Mendeley Data platform (doi: 10.17632/ghcm9z4xhx.1) upon publication.

Detailed additional methods can be found in the supplementary materials.

## Results

### Identification of distinct immune exhaustion subtypes with prognostic significance in DLBCL

To investigate the role of immune exhaustion in DLBCL, we analyzed bulk transcriptomic data from the TCGA NCICCR-DLBCL cohort. The overall study design is illustrated in the flowchart in Supplementary Figure 1A. We utilized 15 well-established immune exhaustion-related genes, selected based on an extensive literature review as canonical markers of T-cell exhaustion and inhibitory checkpoint pathways (leukocyte immunoglobulin-like receptor subfamily B member 1 [*LILRB1*], leukocyte immunoglobulin-like receptor subfamily B member 2 [*LILRB2*], leukocyte associated immunoglobulin like receptor 1 [*LAIR1*], sialic acid binding Ig like lectin 7 [*SIGLEC7*], sialic acid binding Ig like lectin 9 [*SIGLEC9*], V-set immunoregulatory receptor [*VSIR*], CD200 receptor 1 [*CD200R1*], *CD244*, *HAVCR2*, *CD96*, *PDCD1*, *LAG3*, *CTLA4*, *TIGIT*, and B and T lymphocyte associated [*BTLA*]). Unsupervised hierarchical clustering identified two distinct molecular subtypes: high-exhaustion and low-exhaustion groups.

**Figure 1:**
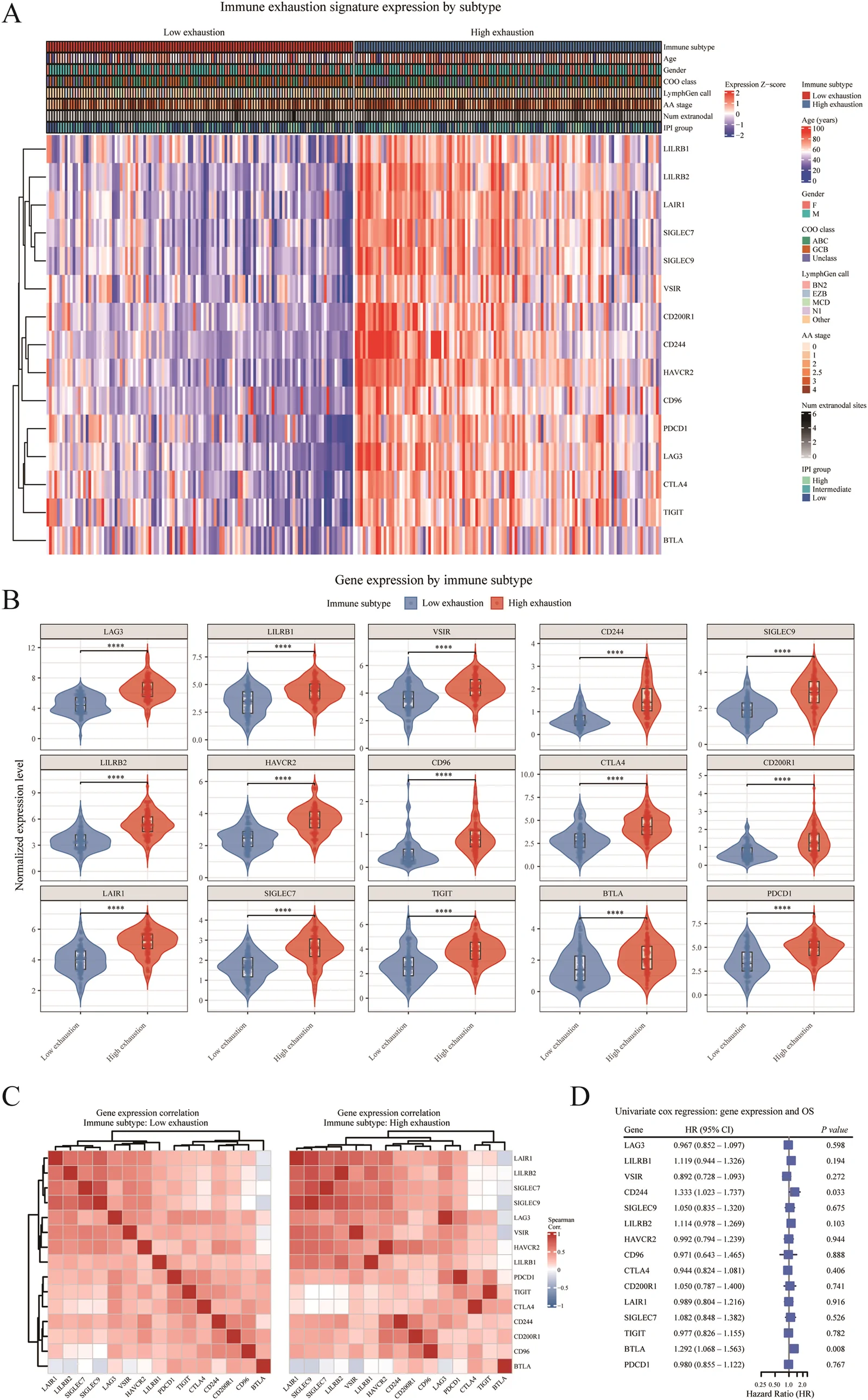
Identification and Characterization of immune exhaustion subtypes in the TCGA-DLBCL Cohort. Heatmap showing the expression of 15 immune exhaustion-related genes in 481 DLBCL patients from the TCGA cohort. Patients are stratified by unsupervised hierarchical clustering into high-exhaustion and low-exhaustion subtypes. Annotations for clinical and molecular features (Age, Gender, COO, LymphGen, AA Stage, IPI) are shown in the tracks above the heatmap. (B) Boxplots comparing the log2-transformed expression levels of each of the 15 genes between the high- and low-exhaustion groups. All comparisons are statistically significant (∗∗∗∗*P* < 0.0001), as determined by the Wilcoxon rank-sum test. (C) Spearman correlation matrices of the 15 genes calculated separately within the high-exhaustion (left) and low-exhaustion (right) subtypes, demonstrating a more coordinated expression program in the high-exhaustion group. The color scale indicates the Spearman correlation coefficient. (D) Forest plot of univariate Cox proportional hazards regression analysis for each of the 15 genes, showing the HR and 95 % CI for overall survival. Genes with a *P-*value <0.05 are highlighted in red. AA stage: Ann Arbor stage; BTLA: B- and T-lymphocyte attenuator; CI: Confidence interval; COO: Cell-of-origin; CTLA4: Cytotoxic T-lymphocyte-associated protein 4; DLBCL: Diffuse large B-cell lymphoma; HAVCR2: Hepatitis A virus cellular receptor 2; HR: Hazard ratio; IL1R1: Interleukin 1 receptor type 1; IL1R2 (Interleukin 1 receptor type 2; IPI: International Prognostic Index; LAIR1: Leukocyte-associated immunoglobulin-like receptor 1; LAG3 (Lymphocyte-activation gene 3; PDCD1: Programmed cell death 1; SIGLEC7: Sialic acid-binding Ig-like lectin 7; SIGLEC9: Sialic acid-binding Ig-like lectin 9; TCGA: The Cancer Genome Atlas; TIGIT: T cell immunoreceptor with Ig and ITIM domains; VSIR: V-set immunoregulatory receptor.

As depicted in Figure 1A, patients in the high-exhaustion subtype exhibited marked and consistent upregulation of all 15 immune exhaustion markers compared to the low-exhaustion subtype. This differential expression pattern represented a distinct biological feature, as other key clinical and molecular characteristics, including patient age, gender, cell-of-origin (COO) class, LymphGen classification, Ann Arbor stage, and International Prognostic Index (IPI) group, were distributed across both exhaustion subtypes without clear clustering. Quantitative analysis confirmed that expression levels of all 15 individual genes were significantly elevated in the high-exhaustion group [Figure 1B], all *P* < 0.0001.

Co-expression analysis revealed strong positive Spearman correlations among the majority of genes within the high-exhaustion group, indicating a highly coordinated and synchronously regulated expression program. In contrast, these correlations were substantially weaker and less consistent in the low-exhaustion group [Figure 1C], suggesting that a robust immune exhaustion-related regulatory network is specifically active in the high-exhaustion subtype. Univariate Cox proportional hazards regression analysis demonstrated that high expression of *BTLA* (hazard ratio [HR] = 1.29, 95% confidence interval [95% CI]: 1.07–1.56, *P* = 0.008) and *CD244* (HR = 1.33, 95 % CI: 1.02–1.74, *P* = 0.033) were significantly associated with poorer overall survival (OS), highlighting these genes as key prognostic markers within the broader immune exhaustion landscape [Figure 1D].

### The high-exhaustion subtype associates with poor clinical outcomes and an inflamed yet dysfunctional tumor microenvironment

**Figure 2:**
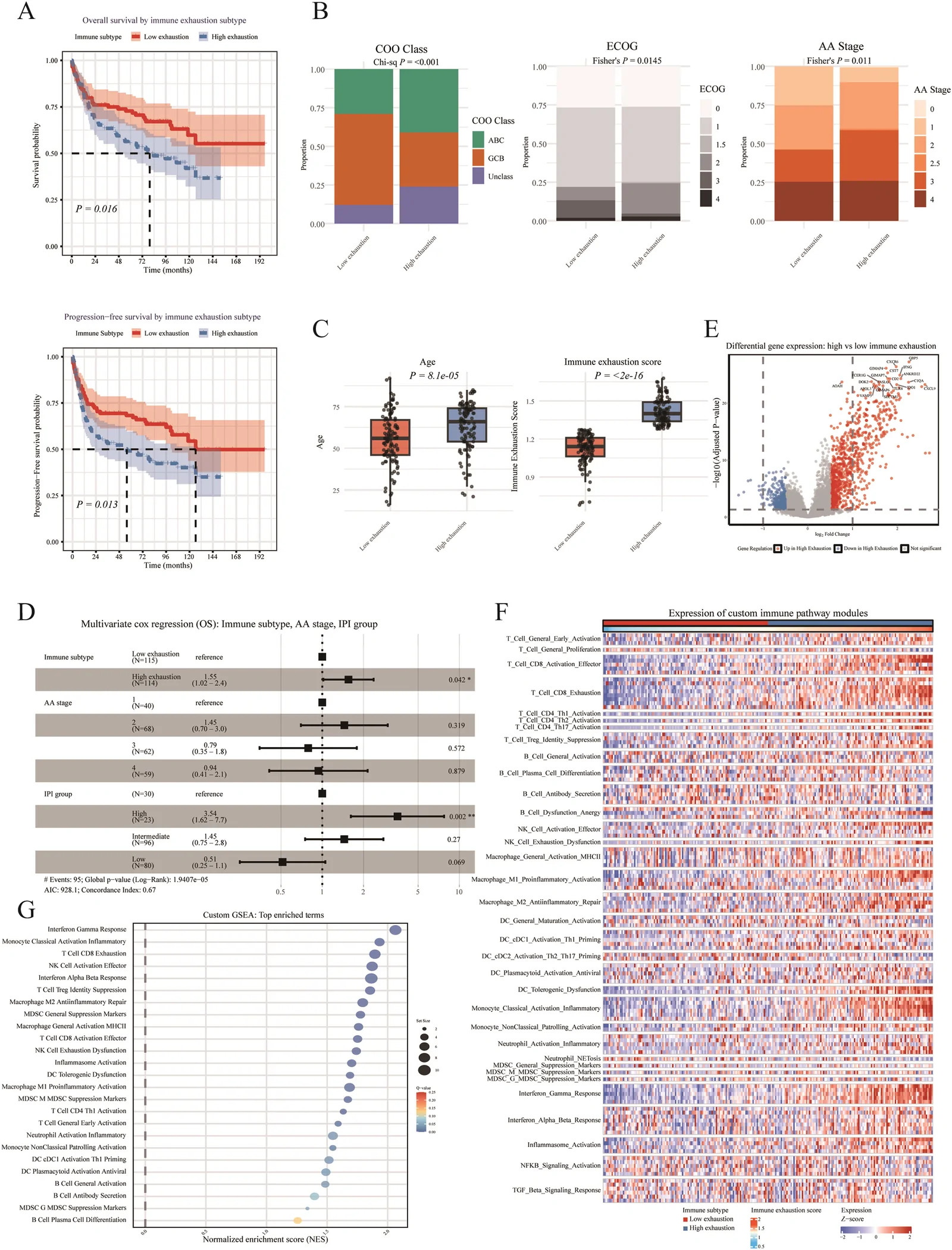
Clinical and Molecular Features of the immune exhaustion subtypes. (A) Kaplan-Meier curves comparing OS (top) and PFS (bottom) between patients in the high- and low-exhaustion subtypes in the TCGA cohort. *P-*values were calculated by the log-rank test. (B) Alluvial plot illustrating the distribution of patients across key clinicopathological features (Age, Gender, COO, Stage, IPI) and the two exhaustion subtypes. (C) Stacked bar plots comparing the distribution of clinical features between the two subtypes, with statistical significance determined by Chi-squared or Fisher's exact tests. The boxplot shows the ssGSEA-based immune exhaustion Score, with significance from a Wilcoxon rank-sum test. (D) Forest plot of a multivariate Cox regression analysis for OS, including the exhaustion subtype, AA stage, and IPI. The model demonstrates that the exhaustion subtype is an independent prognostic factor. (E) Volcano plot showing DEGs between the high- and low-exhaustion groups. Key upregulated immunomodulatory genes are highlighted. (F) Heatmap of ssGSEA scores for 34 custom immune pathway modules, showing distinct pathway activity profiles between the subtypes. (G) GSEA plots for representative hallmark pathways significantly enriched in the high-exhaustion subtype, indicating an inflamed and dysfunctional immune state. AA: Ann Arbor; COO: Cell-of-origin; DEG: Differentially expressed gene; ECOG: Eastern Cooperative Oncology Group performance status; GSEA: Gene Set Enrichment Analysis; IPI: International Prognostic Index; OS: Overall survival; PFS: Progression-free survival; ssGSEA: Single-sample Gene Set Enrichment Analysis; TCGA: The Cancer Genome Atlas.

Having established two distinct immune exhaustion subtypes, we evaluated their clinical significance. Kaplan-Meier analysis revealed that patients in the high-exhaustion group had significantly poorer OS (log-rank *P* = 0.016) and progression-free survival (PFS) (log-rank *P* = 0.013) compared to those in the low-exhaustion group [Figure 2A]. The high-exhaustion subtype was significantly associated with several adverse clinicopathological features, including older age (*P* = 8.1 × 10^−5^), a higher proportion of the more aggressive activated B cell-like (ABC) molecular subtype (Chi-square *P* < 0.001), more advanced Ann Arbor stage (Fisher's *P* = 0.011), and worse Eastern Cooperative Oncology Group (ECOG) performance status (Fisher's *P* = 0.0145) [Figure 2B and C]. Importantly, multivariate Cox proportional hazards regression analysis demonstrated that after adjusting for Ann Arbor stage and IPI group, the high-exhaustion subtype remained an independent predictor of unfavorable OS (HR = 1.55, 95 % CI: 1.02–2.40, *P* = 0.042) [Figure 2D].

To explore the biological basis for these prognostic differences, we performed differentially expressed gene (DEG) analysis between the two subtypes, which revealed widespread transcriptional changes with notable upregulation of key immunomodulatory genes such as C-X-C motif chemokine ligand 9 (*CXCL9*), indoleamine 2,3-dioxygenase 1 (*IDO1*), and interferon gamma (*IFNG*) in the high-exhaustion group (Figure 2E). We calculated an “immune exhaustion Score” for each patient using single-sample Gene Set Enrichment Analysis (ssGSEA) based on the 15 signature genes, which was significantly elevated in the high-exhaustion subtype (*P* < 2 × 10^−16^, Figure 2C). Comprehensive pathway analysis using 34 custom-defined immune modules demonstrated clear distinctions between subtypes, with the high-exhaustion group showing marked upregulation of pathways related to both immune activation and dysfunction [Figure 2F].

Gene Set Enrichment Analysis (GSEA) on the ranked list of DEG confirmed that the high-exhaustion subtype was significantly enriched for pathways indicative of a T-cell-inflamed but exhausted microenvironment. Top enriched pathways included Interferon Gamma Response, Monocyte Classical Activation Inflammatory, T Cell CD8 exhaustion, natural killer (NK) Cell Activation Effector, and Macrophage M1 Proinflammatory Activation [Figure 2G]. Conversely, pathways associated with B cell functions, such as B Cell General Activation and B Cell Antibody Secretion, were enriched in the low-exhaustion group. Collectively, these findings suggest that the poor prognosis of the high-exhaustion subtype is driven by a paradoxically inflamed yet immunosuppressive TME characterized by the co-existence of strong inflammatory signals and potent immune suppressive and exhaustion mechanisms.

### Comprehensive deconvolution reveals distinct cellular composition in the exhausted tumor microenvironment

**Figure 3:**
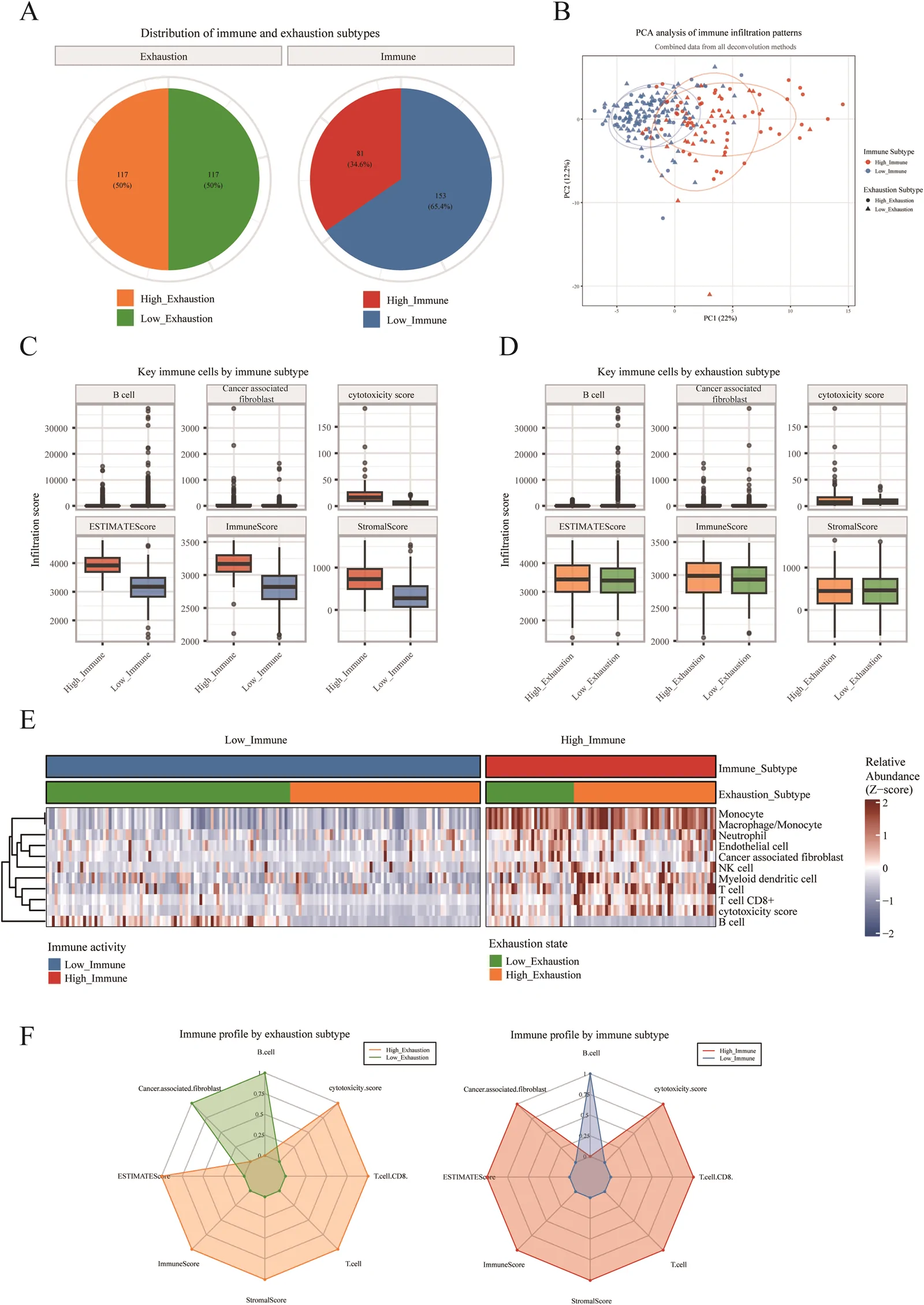
Immune Infiltration Landscape of DLBCL subtypes. (A) Sankey diagram illustrating the patient overlap between the exhaustion subtypes (derived from 15 exhaustion-related genes) and the immune subtypes (derived from k-means consensus clustering of TME deconvolution scores). (B) PCA plot of consensus immune infiltration scores from five deconvolution algorithms. Samples are colored by immune subtype (left) or shaped by exhaustion subtype (right), showing clear separation along the primary axes of variation. (C–D) Boxplots comparing ESTIMATE, immune, Stromal, and other key infiltration scores between immune subtypes (C) and exhaustion subtypes (D). Statistical significance was determined by the Wilcoxon rank-sum test. (E) Heatmap of z-score normalized cell abundance estimates from multiple deconvolution algorithms across all patients, ordered by subtype. This visualization shows coordinated upregulation of multiple immune cell lineages in the high-immune/high-exhaustion groups. (F) Dot plots summarizing and comparing selected immune cell populations between the high- and low-immune/exhaustion subtypes. “T cell CD8^+^” represents cell abundance, while the ‘cytotoxicity score’ reflects a functional signature. ‘Macrophage/Monocyte’ represents a combined population, whereas ‘Monocyte’ refers to a specific cell type as identified by distinct deconvolution algorithms. DLBCL: Diffuse large B-cell lymphoma; PCA: Principal Component Analysis; TME: Tumor microenvironment.

To link the transcriptional subtypes to cellular composition of the TME, we applied five established deconvolution algorithms (xCell, MCP-counter, quantiseq, EPIC, and ESTIMATE) to the TCGA bulk transcriptomic data. Based on consensus immune infiltration profiles, k-means clustering stratified patients into “high-immune” and “low-immune” subtypes [Figure 3A]. A Sankey diagram revealed substantial but incomplete overlap between the high-exhaustion and high-immune groups, suggesting these classifications capture related but distinct biological features. Principal Component Analysis (PCA) of combined infiltration scores from all five methods demonstrated clear separation of patients based on their immune subtype, with the exhaustion-based subtypes also showing separation along the same primary axis [Figure 3B].

Comparative analysis of key immune and stromal cell populations revealed that both the high-immune and high-exhaustion subtypes were characterized by significantly elevated levels of overall immune infiltration, as indicated by higher ESTIMATE, immune, and stromal scores. Specifically, these subtypes showed marked enrichment of T cells, elevated cytotoxicity scores, and increased cancer-associated fibroblasts (CAFs) compared to their “low” counterparts [Figure 3C and D]. Detailed comparison across all five deconvolution methods consistently supported these findings (Supplementary Figure 1B–C), and the different algorithms showed moderate-to-good correlation in their overall predictions, validating our multi-algorithm approach [Supplementary Figure 2A–B].

A comprehensive heatmap of immune and stromal cell abundance, z-score normalized across patients and ordered by immune and exhaustion subtypes, clearly demonstrated that patients in the high-immune group (who were predominantly also in the high-exhaustion group) displayed coordinated upregulation of multiple immune cell lineages [Figure 3E]. This included not only cytotoxic cell types like T cells and NK cells, but also myeloid populations such as monocytes, macrophages, and myeloid dendritic cells. Direct quantitative comparison confirmed that the high-immune subtype exhibited significantly higher infiltration of Macrophage M1, overall Macrophage/Monocyte populations, and T cells, alongside elevated stromal and cytotoxicity scores [Figure 3F and Supplementary Figure 2C]. This integrated analysis characterizes the high-immune/high-exhaustion TME as a highly inflamed, “hot” tumor environment rich in both effector and potentially suppressive myeloid cells, consistent with our GSEA findings.

### Single-cell analysis reveals a dysfunctional TME characterized by exhausted T cells and suppressive B cells

To dissect the cellular heterogeneity and functional states underpinning our bulk-level findings, we analyzed a public single-cell RNA-sequencing (scRNA-seq) dataset of DLBCL patient tumors (GSE182434). We note that this cohort is small (*n* = 4), and the following analysis should be considered exploratory and hypothesis-generating.

**Figure 4:**
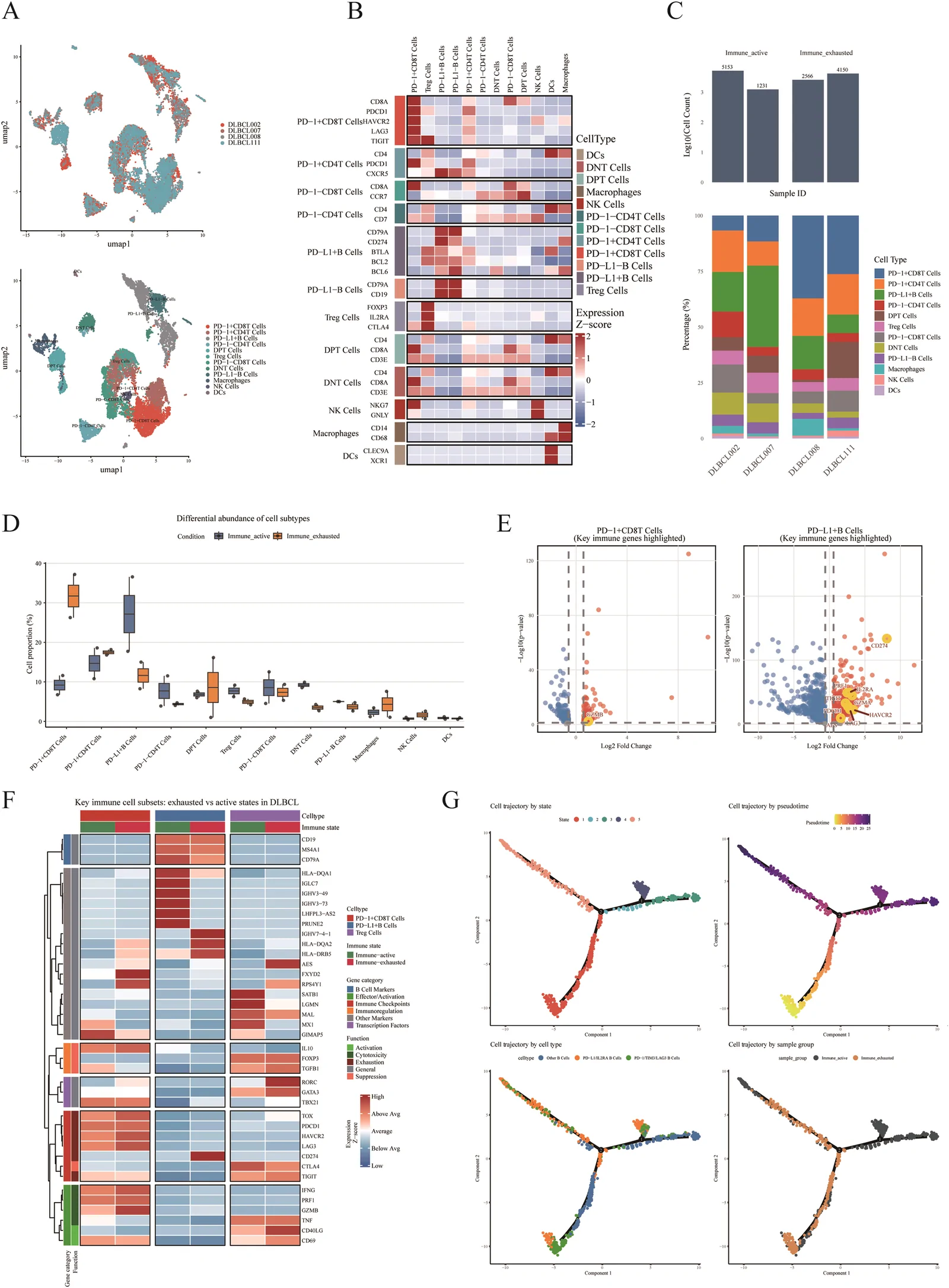
Single-Cell Analysis Defines the Cellular and Molecular Hallmarks of the high-exhaustion Phenotype. (A) UMAP projections of 13,100 immune cells from four DLBCL tumors, colored by individual patient sample (top) and annotated cell type (bottom). (B) Heatmap showing the scaled average expression (Z-score) of canonical marker genes across all identified cell populations, confirming the cell type annotations. (C) Bar plots showing the total number of cells recovered (top, log10 scale) and the relative cellular composition (bottom, percentage) for each patient sample. Samples are grouped into “low-exhaustion” and “high-exhaustion” categories based on their published TME profiles. (D) Bar plot quantifying the log_2_ fold change in the abundance of each cell type when comparing “high-exhaustion” vs. “low-exhaustion” samples. (E) Volcano plots displaying DEGs within PD-1^+^ CD8^+^ T cells (left) and PD-L1^+^ B cells (right), comparing cells from ‘high-exhaustion’ vs. ‘low-exhaustion’ TMEs. (F) Heatmap visualizing the expression of key genes categorized by function across PD-1^+^ CD8^+^ T cells, PD-L1^+^ B cells, and Tregs. Cells are separated by patient group to highlight the coordinated transcriptional upregulation in the “high-exhaustion” state. (G) Pseudotime trajectory analysis of B cells, with individual cells colored by their patient group of origin, suggests a terminal differentiation state for cells from the exhausted TME. DEG: Differentially expressed gene; DLBCL: Diffuse large B-cell lymphoma; NK cells: Natural killer cells; PD-1: Programmed cell death protein 1; PD-L1: Programmed death-ligand 1; scRNA-seq: Single-cell RNA sequencing; TME: Tumor microenvironment; Treg: Regulatory T cell; UMAP: Uniform Manifold Approximation and Projection.

Unsupervised clustering of 13,100 cells identified 12 clusters, including multiple subsets of T cells, B cells, NK cells, macrophages, and dendritic cells [Figure 4A]. Cell type annotations were validated by expression of canonical marker genes, confirming distinct populations such as programmed cell death protein 1 (PD-1^+^) CD8^+^ T cells (*CD8A*, *PDCD1*), programmed death-ligand 1-positive (PD-L1^+^) B cells (*CD79A*, *CD274*), and regulatory T cells (*FOXP3*, *CTLA4*) [Figure 4B].

Based on their TME profiles, we classified the four patients into “high-exhaustion” (*n* = 2) and “low-exhaustion” (*n* = 2) groups [Figure 4C]. While the small sample size limits formal statistical power, this differential abundance analysis revealed a striking enrichment of both PD-1^+^ CD8^+^ T cells and PD-1^+^ CD4^+^ T cells in the “high-exhaustion” samples, accompanied by a significant depletion of the PD-L1^+^ B cell population [Figure 4D]. This suggests a potential, though technically challenging, hallmark of the “high-exhaustion” TME-an expansion of antigen-experienced T cells alongside contraction of a key PD-L1-expressing B cell subset.

Differential gene expression analysis comparing cells from the “high-exhaustion” vs “low-exhaustion” TME revealed a molecular signature characteristic of classical immune exhaustion. To ensure these findings were not driven by technical artifacts such as doublets, we applied rigorous filtering using scDblFinder. Within the accumulated PD-1^+^ CD8^+^ T cell population, key genes associated with cytotoxic effector function, such as granzyme B (*GZMB*), were significantly upregulated in the “high-exhaustion” TME [Figure 4E, left panel], suggesting these cells are receiving activation signals and attempting to mount a cytotoxic response. More dramatically, within the PD-L1^+^ B cell population, a broad suite of crucial immune-related genes was significantly upregulated, including the defining immunosuppressive ligand *CD274* (PD-L1) itself, multiple T-cell-associated checkpoint molecules (*PDCD1*, *TIGIT*, *LAG3*, *HAVCR2*), and cytotoxic genes (perforin 1 [*PRF1*], granzyme A [*GZMA*]) [Figure 4E, right panel].

This coordinated transcriptional signature was further substantiated by comprehensive heatmap analysis visualizing expression of key functional gene categories across PD-1^+^ CD8^+^ T cells, PD-L1^+^ B cells, and regulatory T cells. The heatmap revealed a clear and consistent pattern: in the “high-exhaustion” TME, there was coordinated upregulation of genes within the effector/activation, immune checkpoint, and immunoregulation categories across all three cell lineages compared to their counterparts in the “low-exhaustion” TME [Figure 4F]. This coordinated upregulation of both pro-inflammatory and inhibitory pathways confirms that the “high-exhaustion” TME represents a state of active yet dysfunctional immune engagement rather than immune ignorance or quiescence.

To model the dynamic progression towards this exhausted state, we performed pseudotime trajectory analysis on B cells. The analysis reconstructed a continuous cellular trajectory, and when mapping cells from the two patient groups onto this path, cells from the “high-exhaustion” samples were predominantly located at the terminal end of the pseudotime trajectory [Figure 4G], suggesting that the exhausted phenotype represents the endpoint of a differentiation or exhaustion process-a state of terminal dysfunction.

### Development and Validation of a five-gene Immune Exhaustion-Related Prognostic Score

Having identified a biologically distinct and prognostically relevant high-exhaustion subtype, we next aimed to develop a quantitative and clinically applicable gene signature-the IERPS-to capture this phenotype.

To translate our findings into a clinically applicable tool, we employed LASSO-Cox regression analysis on the GSE181063 discovery cohort to identify the most robust prognostic genes from the initial 15-gene signature. This procedure identified a five-gene signature composed of *LILRB2*, *CD96*, *LAIR1*, *BTLA*, and *PDCD1*. Based on the expression levels of these genes and their weighted LASSO-Cox coefficients, we established an IERPS calculated as: IERPS = (0.1280 × *LILRB2*) + (−0.4589 × CD96) + (0.6940 × *LAIR1*) + (0.0144 × BTLA) + (0.0493 × *PDCD1*). Notably, four genes (*LILRB2*, *LAIR1*, *BTLA*, *PDCD1*) contributed positively to the risk score, consistent with their roles as inhibitory receptors, while CD96 exhibited a protective coefficient. The proportional hazards assumption was tested and met for the final model.

**Figure 5:**
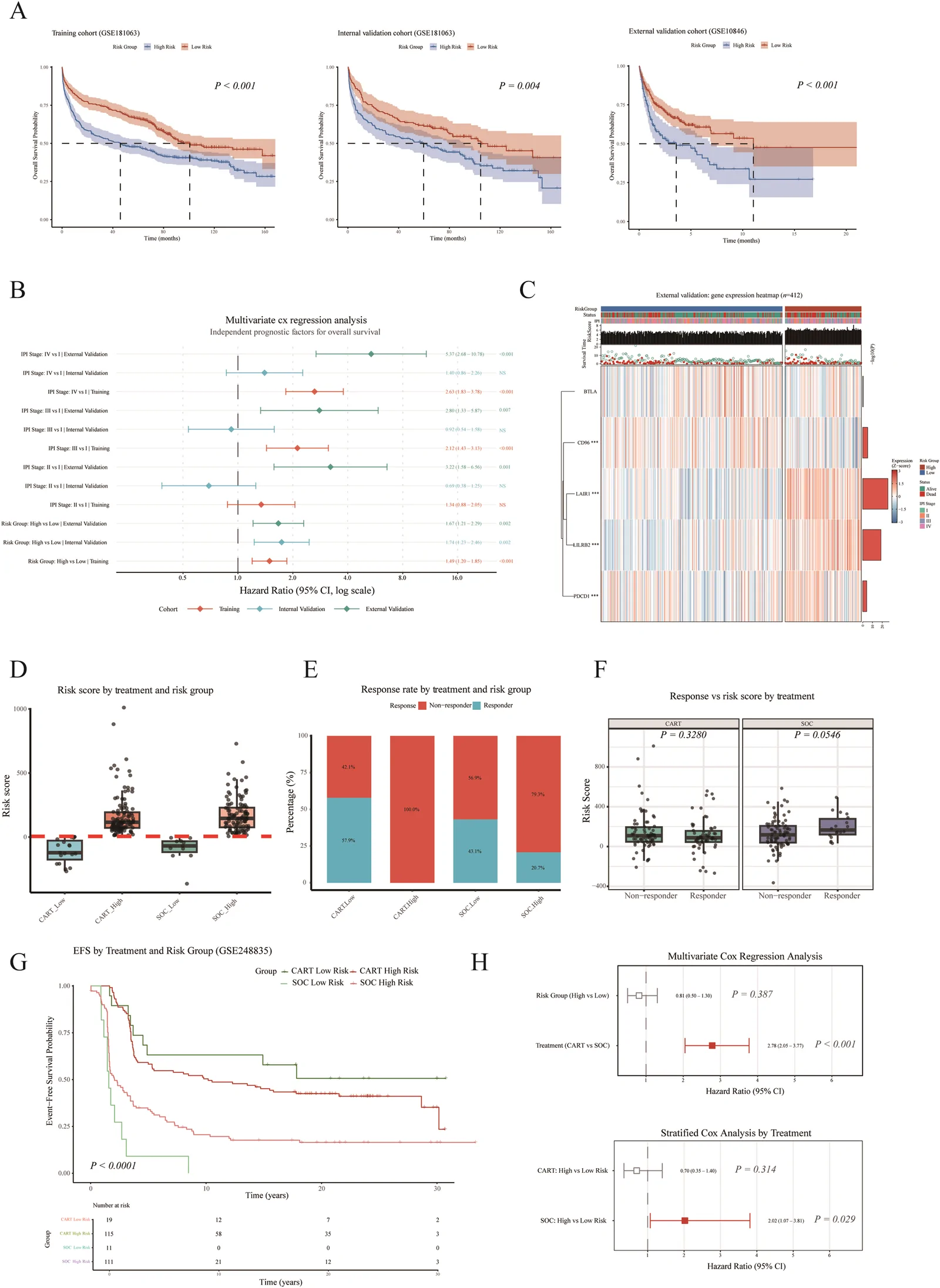
Development and Validation of the IERPS. (A) Kaplan-Meier curves for overall survival based on IERPS stratification (high-vs. low-risk) in the discovery cohort (GSE181063 training set, left), internal validation cohort (GSE181063 test set, middle), and external validation cohort (GSE10846, right). *P-*values were calculated using the log-rank test. (B) Forest plot of multivariate Cox regression analysis for OS in all three cohorts, demonstrating that IERPS is an independent prognostic factor after adjusting for IPI stage. (C) Heatmap showing the expression of the five IERPS genes, patient survival status, and calculated IERPS values in the external validation cohort. (D) Boxplots showing the distribution of the continuous IERPS (Risk Score) across the four subgroups defined by treatment arm (CAR-T and SOC) and IERPS-stratified risk group (low and high) in the GSE248835 R/R DLBCL cohort. (E) Bar plot of treatment response rates (Complete or Partial Response) in the SOC and CAR-T arms, stratified by IERPS risk group. (F) Boxplots comparing IERPS values between responders and non-responders in each treatment arm. Statistical significance was determined by the Wilcoxon rank-sum test. (G) Kaplan-Meier curves for EFS for the four subgroups. A formal interaction test confirms that the prognostic effect of IERPS is significantly different between the two treatment arms (Interaction *P* < 0.0001). (H) Forest plot of stratified Cox regression analysis for EFS, showing the HR for the IERPS risk group calculated separately within the SOC and CAR-T arms. CAR-T: Chimeric antigen receptor T-cell; DLBCL: Diffuse large B-cell lymphoma; EFS: Event-free survival; HR: Hazard ratio; IERPS: immune exhaustion-Related Prognostic Score; IPI: International Prognostic Index; OS: Overall survival; R/R: Relapsed/refractory; SOC: Standard-of-care. ***P < 0.0001.

We evaluated the prognostic performance of the IERPS across multiple independent cohorts using the median IERPS value from the discovery cohort as a universal cutoff to dichotomize patients into high-risk and low-risk groups. Time-dependent receiver operating characteristic (ROC) curves were constructed to quantify the time-dependent predictive capacity of the IERPS at 12, 36, and 60 months across training, internal and external validation cohorts [Supplementary Figure 3A -B]. Kaplan-Meier analysis demonstrated that high-risk patients had significantly shorter OS compared to low-risk patients in the discovery cohort (*P* < 0.001), internal validation cohort (*P* = 0.004), and external validation cohort GSE10846 (*P* < 0.001) [Figure 5A].

To determine whether the IERPS provided prognostic information beyond established clinical risk factors, we performed multivariate Cox regression analysis adjusting for the IPI stage. The IERPS-defined risk group remained a strong and statistically significant independent predictor of poor OS across all three cohorts: discovery (HR = 1.49, *P* < 0.001), internal validation (HR = 1.74, *P* = 0.002), and external validation (HR = 1.67, *P* = 0.002) [Figure 5B]. A heatmap visualizing the five signature genes in the external validation cohort confirmed the model's consistency: patients with higher IERPS values exhibited increased mortality and elevated expression of the four risk-contributing genes (*LILRB2*, *LAIR1*, *BTLA*, *PDCD1*) alongside decreased expression of the protective gene *CD96* [Figure 5C]. Importantly, the multivariate Cox analysis confirmed that IERPS remained a significant and independent predictor of poor prognosis even after adjusting for the IPI [Figure 5B], highlighting its added clinical value.

### The IERPS identifies high-risk patients who overcome poor prognosis through CAR-T cell therapy

We next investigated the clinical utility of the IERPS in a cohort of R/R DLBCL patients (GSE248835) treated with either SOC or CAR-T cell therapy [Figure 5D]. We first assessed the model's ability to predict treatment response. In the SOC arm, patients in the high-risk group had a markedly lower response rate (20.7 %) compared to low-risk patients (43.1 %), whereas this association was not observed in the CAR-T arm, where response rates were high regardless of risk status (79.3 % vs 100.0 %) [Figure 5E]. Consistently, the continuous IERPS showed a non-significant trend toward higher in non-responders within the SOC arm (*P*=0.0546) but not in the CAR-T arm (*P*=0.328). [Figure 5F].

Most importantly, we evaluated the model's ability to predict event-free survival (EFS) in the context of these different treatments. Kaplan-Meier analysis of all four subgroups revealed a dramatic interaction between the IERPS-defined risk group and treatment modality (Interaction *P* < 0.0001) [Figure 5G]. While patients in the SOC high-risk group had the worst prognosis, CAR-T cell therapy appeared to largely attenuate the poor prognosis associated with a high IERPS, with the survival curve for high-risk patients receiving CAR-T becoming nearly indistinguishable from that of low-risk patients. Stratified survival analyses confirmed this pattern: the IERPS effectively stratified patients in the SOC cohort (*P* = 0.026) but lost its prognostic significance in the CAR-T cohort (*P* = 0.310) [Supplementary Figure 4A–B].

Stratified Cox regression analysis quantified this interaction, confirming that while the IERPS was strongly prognostic in the SOC arm (HR for high-risk vs low-risk = 2.02, 95%CI: 1.07–3.81, *P* = 0.029), it was not prognostic in the CAR-T arm (HR = 0.70, *P* = 0.314) [Figure 5H]. These findings strongly suggest that the IERPS not only identifies high-risk DLBCL patients but also specifically pinpoints a population that derives disproportionate benefit from CAR-T cell therapy over SOC.

### The IERPS dynamically tracks clinical response to T-cell engaging bispecific antibody therapy

To explore the potential of the IERPS as a dynamic biomarker, we performed a proof-of-concept analysis on an in-house longitudinal cohort of two DLBCL patients treated with Columvi (glofitamab), a CD20 × CD3 bispecific antibody. Peripheral blood samples were collected at three timepoints-pre-treatment (Pre), during-treatment (During), and post-treatment (Post)-and subjected to bulk RNA seq.

**Figure 6:**
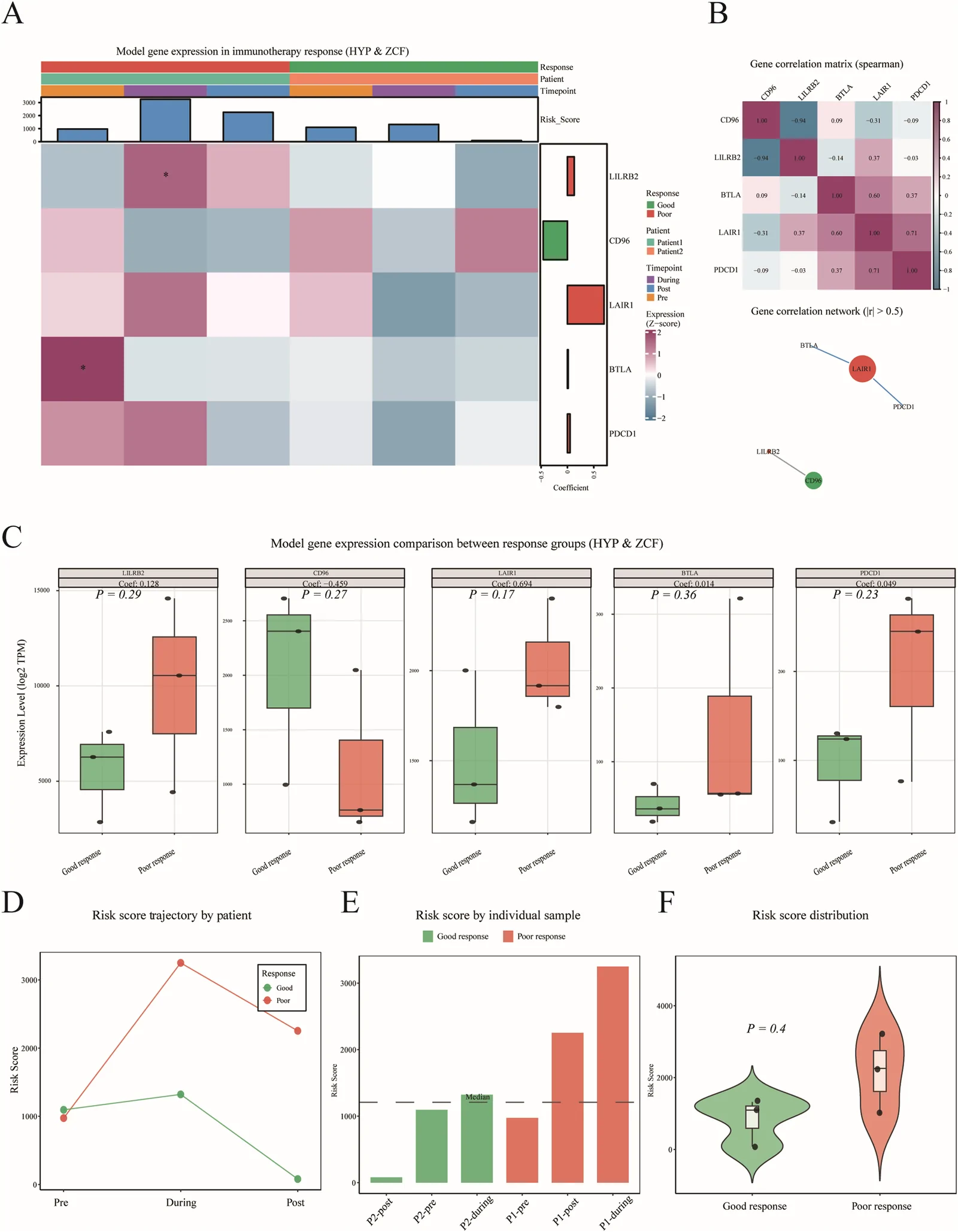
The IERPS Dynamically Tracks Response to Columvi (Glofitamab). (A) Integrated heatmap showing the expression of the five IERPS genes, their model coefficients, and the calculated IERPS values for six longitudinal peripheral blood samples from a poor responder (Patient 1) and a good responder (Patient 2). (B) Spearman correlation matrix of the five signature genes across the six samples. (C) Boxplots comparing the expression of individual IERPS genes between samples from the good- and poor-response patients. (D) Line plot showing the IERPS trajectory over three timepoints (Pre-, During-, Post-treatment) for each patient. (E) Bar plot showing the IERPS for each of the six individual samples. (F) Boxplot comparing the IERPS values aggregated by patient outcome. CAR-T: Chimeric antigen receptor T-cell; DLBCL: Diffuse large B cell lymphoma; IERPS: immune exhaustion-Related Prognostic Score; LAIR1: leukocyte associated immunoglobulin like receptor 1; LILRB2: leukocyte immunoglobulin like receptor B2; PDCD1: programmed cell death 1; R/R: Relapsed/refractory; TME: Tumor microenvironment.

An integrated heatmap visualizing the expression of the five model genes, their corresponding model coefficients, and calculated IERPS for all six samples revealed distinct molecular and risk score profiles between the two patients [Figure 6A]. The poor-response patient (Patient 1) consistently displayed higher IERPS values driven by elevated expression of major risk-contributing genes like *LAIR1* and reduced expression of the protective gene *CD96*, whereas the good-response patient (Patient 2) exhibited lower IERPS associated with sustained high *CD96* expression and lower levels of risk-associated genes.

Correlation analysis suggested that the co-expression relationships observed in larger cohorts were preserved in this small dataset [Figure 6B]. The risk-associated genes (*LAIR1*, *BTLA*, *PDCD1*) demonstrated strong positive correlations among themselves, while *CD96* showed negative correlations with several risk genes, reinforcing the biological consistency of the signature. Analysis of individual gene expression between outcome groups showed that expression trends aligned with the genes' roles in the model: the protective gene *CD96* tended to be higher in the good responder, whereas the high-risk gene *LAIR1* was consistently elevated in the poor responder across all timepoints [Figure 6C].

This exploratory analysis suggests that the IERPS trajectory over the treatment course demonstrated opposing dynamic trends. The score for the poor responder (Patient 1) remained elevated throughout treatment, while the score for the good responder (Patient 2) started lower and progressively declined, reaching its nadir post-treatment [Figure 6D and E]. When summarized by overall clinical outcome, samples from the good responder consistently clustered at a lower IERPS than those from the poor responder [Figure 6F]. While statistical conclusions cannot be drawn from two patients, this descriptive observation provides preliminary evidence that the IERPS may dynamically reflect the underlying immune state of patients undergoing T-cell-engaging therapy, offering potential for real-time insights into treatment efficacy.

## Discussion

In this study, we dissected the immune landscape of DLBCL, defining its adverse phenotype as a state of classical T-cell exhaustion within a suppressive TME, and developed a clinically powerful biomarker with both prognostic and predictive capabilities.

The IERPS model provides a focused, quantitative measure of a specific biological axis—T-cell exhaustion—which complements broader classification systems like the COO (ABC/germinal center B cell-like [GCB]) or LymphGen molecular subtypes. While these standard systems capture the intrinsic biology of the tumor cell, the IERPS specifically quantifies the functional state of the immune microenvironment. It likely overlaps with the ‘T-cell inflamed’ yet non-responsive phenotypes described in other studies, but provides a simple, clinically applicable 5-gene score specifically optimized for DLBCL.

Our primary biological finding clarifies the nature of the dysfunctional TME in DLBCL. Our exploratory single-cell analysis revealed that the “high-exhaustion” state is not quiescent but is characterized by an active, yet futile, immune response. The upregulation of GZMB in PD-1^+^ T cells, coupled with the broad expression of multiple inhibitory checkpoints on surrounding B cells (PD-L1, *TIGIT*, *LAG3*), paints a clear picture of classical T-cell exhaustion.^6^^,^^7^ A particularly striking finding from our exploratory single-cell analysis was the co-expression of T-cell-associated checkpoint and cytotoxic genes within the PD-L1^+^ B cell population. We acknowledge that such patterns can sometimes arise from technical artifacts, specifically “doublets”. However, our bioinformatic pipeline included a rigorous filtering step using scDblFinder to computationally identify and remove these artifacts prior to analysis. Therefore, we posit a biological explanation: The B cells themselves appear to adopt a regulatory B cell (Breg)-like, suppressive phenotype, contributing actively to this TME dysfunction.^14^ Nevertheless, given the small sample size of our single-cell cohort (*n* = 4), this hypothesis regarding B cell plasticity remains exploratory and requires direct functional validation. We successfully translated this biological insight into a robust five-gene IERPS model. The IERPS serves as a quantitative surrogate for this exhausted TME state. Interestingly, the IERPS model assigned a negative (protective) coefficient to *CD96*, despite its higher expression in the high-exhaustion subtype. This highlights the important distinction between simple differential expression and multivariate prognostic modeling. While *CD96* is clearly part of the exhaustion landscape, the LASSO model suggests that, when considered alongside other powerful inhibitory receptors like *LILRB2* and *LAIR1*, its relative expression level has a counteracting prognostic influence. This may reflect context-dependent roles of checkpoint molecules or potentially suggests that *CD96* marks a subset of exhausted cells that retain some residual functional capacity. Its ability to act as an independent prognostic factor across multiple large cohorts, outperforming established tools like the IPI, highlights its clinical relevance in capturing this crucial biological axis of DLBCL.^3^

Our IERPS model provides a focused, quantitative measure of a specific biological axis-T-cell exhaustion-which complements broader classification systems like the cell-of-origin (ABC/GCB) or LymphGen molecular subtypes. For instance, the LymphGen ST2 subtype is also characterized by an immune-infiltrated TME. However, ST2 is a broad classification based on stromal and immune response signatures, whereas the IERPS is a more specific, functionally oriented score optimized to quantify the degree of T-cell dysfunction. This specificity is likely key to its predictive power for CAR-T cell therapy.^15^^,^^16^ While a general ‘T-cell inflamed’ signature may predict response to checkpoint inhibitors, the IERPS captures a state of profound endogenous T-cell failure that is specifically bypassed by the engineered effectors of CAR-T cell Therapy. Therefore, IERPS is not merely another prognostic immune signature; it is a predictive tool tailored to a specific therapeutic modality.

The most significant finding of our study is the profound interaction between the IERPS and CAR-T cell Therapy efficacy. The model's predictive power does not lie in identifying CAR-T resistance; on the contrary, CAR-T cell therapy was highly effective across both risk groups. Instead, the IERPS identifies patients for whom CAR-T cell therapy is most transformative. CAR-T cell therapy completely abrogated the dismal prognosis of IERPS-high patients, elevating their survival to that of the IERPS-low group. This provides a strong mechanistic rationale: the IERPS-high state reflects an ‘Intrinsic immune Dysfunctional’ subtype, where the endogenous T-cell compartment is exhausted, and which conventional therapies fail to rescue. CAR-T cell therapy succeeds by acting as a functional immune reconstitution strategy, bypassing this compromised endogenous T-cell machinery entirely and replacing it with potent, engineered anti-tumor effectors. Further reinforcing the biological relevance of the IERPS, our longitudinal analysis of patients treated with the bispecific antibody glofitamab provided complementary evidence. The ability of the score to dynamically track with clinical response demonstrates that the IERPS reflects the real-time state of the endogenous immune system as it is being therapeutically engaged.^12^ While this analysis is descriptive due to the small sample size (*n* = 2), it serves as a proof-of-concept that suggests its potential utility as a pharmacodynamic biomarker, distinct from its pre-treatment predictive role for CAR-T.

Regarding clinical translation, we recognize that the IERPS is currently calculated based on RNA-seq data, which may limit rapid adoption in primary clinical settings. To improve accessibility, future efforts should focus on developing standardized detection methods, such as a custom reverse transcription quantitative polymerase chain reaction (RT-qPCR) panel or a NanoString nCounter assay based on these five genes. These platforms offer better cost-effectiveness and faster turnaround times than whole-transcriptome sequencing, facilitating integration into routine clinical workflows. Ultimately, the validation of IERPS as a potential predictive biomarker requires prospective testing. A future clinical trial could enroll R/R DLBCL patients, measure IERPS from pre-treatment tumor biopsies, and use it as a stratification factor to test its utility in guiding the choice between CAR-T cell therapy and other novel agents. Positioning IERPS as a pre-specified exploratory biomarker in such trials is the critical next step to confirm its clinical utility. Our study has several limitations. First, the majority of analyses are retrospective. Second, we establish a strong predictive association but do not directly probe the causal mechanisms; analyzing paired pre- and post-CAR-T cell therapy samples would be an ideal approach to understand how CAR-T cells remodel the IERPS-high TME, but suitable public datasets were not available for this analysis. Third, the lack of consistent COO (ABC/GCB) annotation across public cohorts prevented a formal stratified analysis. Finally, while the predictive value of IERPS for CAR-T cell therapy was demonstrated in a large cohort, validation in a second, fully independent cohort of CAR-T-treated patients is a crucial next step to confirm its generalizability. Future prospective studies are needed to validate the IERPS's predictive value in guiding treatment decisions for R/R DLBCL in a clinical trial setting.

In summary, we define the adverse immune phenotype in DLBCL as a state of classical T-cell exhaustion within a suppressive TME. Our validated five-gene IERPS model robustly captures this dysfunctional state, identifying patients with poor prognosis under standard therapy. Critically, the IERPS functions as a potential predictive biomarker, pinpointing high-risk patients who can overcome this poor prognosis specifically through treatment with CAR-T cell therapy. The IERPS represents a promising and clinically actionable tool to advance personalized immunotherapy strategies in DLBCL.

## Authors contribution

Conceptualization: Bo Zheng, Rong Li; Methodology: Bo Zheng, Wanqian Hu, Ying Fang; Writing – original draft, formal analysis, and investigation: Bo Zheng; Data Curation: Bo Zheng, Wanqian Hu; Writing – review & editing, supervision, and funding acquisition: Rong Li. All the authors critically revised and approved the final version of the manuscript.

## Ethics statement

The studies involving human participants were conducted in accordance with the ethical standards of the Institutional Review Board of Naval Medical Center on human experimentation and with the *Helsinki Declaration* of 1975, as revised in 2000. The majority of this study was based on the analysis of publicly available, de-identified patient data from The Cancer Genome Atlas (TCGA) and Gene Expression Omnibus (GEO) databases. The collection of samples for the in-house longitudinal cohort was performed under a clinical protocol (No. AF-HEC-033) approved by the Institutional Review Board of the Navy Medical Center of PLA (No. AF-HEC-033), and written informed consent was obtained from both patients involved.

## Data availability statement

The datasets used in this study are publicly available from TCGA and GEO. All analysis code used to generate the results and figures will be made available on the Mendeley Data platform (doi: 10.17632/ghcm9z4xhx.1).

## Declaration of generative AI and AI-assisted technologies in the writing process

The authors declare that generative artificial intelligence (AI) and AI-assisted technologies were not used in the writing process or any other process during the preparation of this manuscript.

## Funding

This study was supported by the 10.13039/501100001809National Natural Science Foundation of China (No. 82300235), the Shanghai Municipal Health Commission Talent Plan Youth Project (No. 2022YQ031), and the Sanhang Talent Program of Naval Medical University (No. 25TPSL0003).

## Conflict of interest

The authors declare that they have no known competing financial interests or personal relationships that could have appeared to influence the work reported in this paper.
