# Supplementary material for: An immune exhaustion signature predicts prognosis and identifies patients with diffuse large B-cell lymphoma (DLBCL) who derive preferential benefit from chimeric antigen receptor (CAR)-T cell therapy
Source: Cancer Pathog Ther. 2026 Feb 13;4(5):421–33. doi: 10.1016/j.cpt.2026.02.002 (PMC13559917; doi:10.1016/j.cpt.2026.02.002)
Supplement: Supplementary file 1 [file mmc1.docx]

**Supplementary data**

**Supplementary legends:**

**Supplementary Figure 1. (A) Flowchart of the overall study design. (B)** Boxplots comparing key immune and stromal cell scores between the High-Immune and Low-Immune subtypes, as estimated by each of the five individual deconvolution algorithms (xCell, MCP-counter, quantiseq, EPIC, and ESTIMATE). **(C)** Corresponding boxplots comparing the same scores between the high-exhaustion and low-Exhaustion subtypes. Statistical significance was determined by the Wilcoxon rank-sum test.

**Supplementary Figure 2. (A)** Heatmap showing the Spearman's rank correlation matrix of immune infiltration scores estimated by the five different deconvolution algorithms. Higher correlation coefficients (indicated by darker colors) demonstrate good concordance between methods. **(B)** Box plot showing the distribution of raw infiltration scores for each of the five deconvolution methods (EPIC, ESTIMATE, MCP-counter, quantiseq, and xCell) across all patients. **(C)** A comprehensive set of boxplots comparing the infiltration scores of all measured immune and stromal cell populations between the High-Immune and Low-Immune subtypes. Statistical significance was determined by the Wilcoxon rank-sum test.

**Supplementary Figure 3. (A) Time-dependent receiver operating characteristic (ROC) curves for the** Immune Exhaustion-Related Prognostic Score (**IERPS) in the training and validation cohorts, showing** area under the curve (AUC) **values over time. (B) Distribution of C-index values indicating the discriminatory power of the IERPS model.**

**Supplementary Figure 4. (A)** Kaplan-Meier curve showing event-free survival (EFS) for patients stratified by the Immune Exhaustion-Related Prognostic Score (**IERPS)**(High- vs. low-risk) within the **Standard-of-Care (SOC) arm only**. The IERPS demonstrates significant prognostic value in this subgroup. **(B)** Kaplan-Meier curve showing EFS for patients stratified by the IERPS within the **CAR-T cell Therapy arm only**. *P*-values were calculated using the log-rank test.

**Supplementary materials and methods**

**1. Functional and Pathway Enrichment Analysis**

Differential gene expression (DGE) analysis between the High- and low-Exhaustion subtypes was conducted using the DESeq2 R package, with a significance threshold of |log2FoldChange| > 1 and an adjusted *P*-value < 0.05.

Single-sample Gene Set Enrichment Analysis (ssGSEA) was performed using the GSVA R package to calculate an "Immune Exhaustion Score" for each patient based on the 15-gene signature. Additionally, ssGSEA was used to quantify the activity of 34 custom-defined immune pathway modules (Supplementary Table 1).

Pre-ranked Gene Set Enrichment Analysis (GSEA) was performed using the fgsea R package. Genes were ranked based on the test statistic from the DGE analysis. Enrichment was tested against custom and MSigDB (v7.4) hallmark gene sets.

**2. Tumor Microenvironment (TME) Deconvolution**

The cellular composition of the TME was estimated from bulk transcriptomic data using a panel of five established algorithms: xCell, MCP-counter, quantiseq, EPIC, and ESTIMATE. The resulting infiltration scores were combined, and k-means consensus clustering was applied to stratify patients into "High-Immune" and "Low-Immune" subtypes. Principal Component Analysis (PCA) was performed on the combined infiltration scores to visualize the main axes of variation.

**3. Single-Cell RNA-Sequencing (scRNA-seq) Data Analysis**

The pre-processed scRNA-seq count matrix was analyzed using the Seurat R package (v4.3.0). Standard quality control was applied. To minimize technical artifacts, putative doublets were identified and removed using the scDblFinder package prior to downstream analysis. The data was normalized using NormalizeData, and variable features were identified with FindVariableFeatures. The data was scaled, and dimensionality was reduced using PCA. Cell clusters were identified using the FindNeighbors and FindClusters functions. Cell types were manually annotated based on the expression of canonical marker genes.

Differential cell abundance analysis between the "Immune-exhausted" and "Immune-active" groups was performed. Differential gene expression analysis within specific cell populations between the two groups was conducted using the FindMarkers function with the Wilcoxon rank-sum test. Pseudotime trajectory analysis was performed using the Monocle 2 package to model cellular differentiation states.

**4. Development and Validation of the Immune Exhaustion-Related Prognostic Score (IERPS) Model**

The GSE181063 cohort was randomly partitioned into a training set (70% of patients) and an internal validation set (30%). In the training set, a LASSO-Cox proportional hazards regression model was built using the glmnet R package to select the most prognostically relevant genes from the initial 15-gene list. The optimal penalty parameter (lambda) was determined via 10-fold cross-validation based on the minimum mean cross-validated error. The proportional hazards assumption was tested and met for the final model.

The IERPS was calculated for each patient using a linear combination of the expression levels of the selected genes, weighted by their respective LASSO-Cox coefficients. The formula is as follows:

*IERPS = Σ (Coef_i × Expr_i)*

where Coef_i is the coefficient for gene i, and Expr_i is its log2-transformed expression level.

Patients in all cohorts were dichotomized into high-risk and low-risk groups using the median IERPS value calculated from the training set as the universal cutoff. This fixed cutoff was applied to the internal validation set, the external validation set (GSE10846), and the CAR-T cell Therapy cohort (GSE248835).

**5. Statistical Analysis**

All statistical analyses and visualizations were performed using R software (version 4.2.1) and relevant packages. Survival curves were generated using the Kaplan-Meier method and compared with the log-rank test. Univariate and multivariate Cox proportional hazards regression analyses were used to calculate hazard Ratios (HR) and assess the independent prognostic value of the IERPS. An interaction term (IERPS_risk_group, Treatment_arm) was included in the Cox model to test for a differential treatment effect in the chimeric antigen receptor T-cell (CAR-T) cohort.

Comparisons of continuous variables between two groups were performed using the two-sided Wilcoxon rank-sum test or Student's *t*-test. Proportions were compared using the Chi-squared test or Fisher's exact test. A *P*-value < 0.05 was considered statistically significant. For multiple comparisons, *P*-values were adjusted where appropriate.

**Supplementary Table 1 34 custom-defined immune pathway modules**

| **Signature Name** | **Genes** |
| --- | --- |
| **T Cell Signatures** |  |
| T_Cell_General_Early_Activation | *CD69, IL2RA, CD44* |
| T_Cell_General_Proliferation | *MKI67* |
| T_Cell_CD8_Activation_Effector | *GZMB, PRF1, IFNG, LAMP1, TNF* |
| T_Cell_CD8_Exhaustion | *PDCD1, CTLA4, HAVCR2, LAG3, TIGIT, TOX, ENTPD1, EOMES* |
| T_Cell_CD4_Th1_Activation | *IFNG, TBX21, TNF, IL2* |
| T_Cell_CD4_Th2_Activation | *IL4, IL5, IL13, GATA3* |
| T_Cell_CD4_Th17_Activation | *IL17A, IL17F, IL22, RORC, IL21* |
| T_Cell_Treg_Identity_Suppression | *FOXP3, IL2RA, CTLA4, IKZF2, TNFRSF18, IL10* |
| **B Cell Signatures** |  |
| B_Cell_General_Activation | *CD69, CD80, CD86, AICDA* |
| B_Cell_Plasma_Cell_Differentiation | *PRDM1, SDC1, XBP1, IRF4* |
| B_Cell_Antibody_Secretion | *JCHAIN, IGHG1, IGHA1, IGLC2, IGKC* |
| B_Cell_Dysfunction_Anergy | *FCER2, FASLG, CD22* |
| **NK Cell Signatures** |  |
| NK_Cell_Activation_Effector | *NCR1, NCR3, KLRK1, GZMB, PRF1, IFNG, LAMP1, FCGR3A* |
| NK_Cell_Exhaustion_Dysfunction | *PDCD1, HAVCR2, LAG3, TIGIT, KLRC1* |
| **Macrophage Signatures** |  |
| Macrophage_General_Activation_MHCII | *HLA-DRA, HLA-DRB1, HLA-DPA1, HLA-DPB1, CIITA* |
| Macrophage_M1_Proinflammatory_Activation | *NOS2, TNF, IL1B, IL6, CD86, CXCL9, CXCL10, IRF5* |
| Macrophage_M2_Antiinflammatory_Repair | *MRC1, ARG1, IL10, CD163, TGFB1, CCL18, CCL22, IRF4* |
| **Dendritic Cell (DC) Signatures** |  |
| DC_General_Maturation_Activation | *CD80, CD83, CD86, CCR7, LAMP3* |
| DC_cDC1_Activation_Th1_Priming | *IL12B, IRF8, BATF3, XCR1, CLEC9A* |
| DC_cDC2_Activation_Th2_Th17_Priming | *IRF4, CD1C, FCER1A, IL23A* |
| DC_Plasmacytoid_Activation_Antiviral | *IL3RA, CLEC4C, NRP1, TCF4, IRF7, IFNA1, IFNB1* |
| DC_Tolerogenic_Dysfunction | *IL10, IDO1, TGFB1, PDCD1LG1, PDCD1LG2* |
| **Monocyte Signatures** |  |
| Monocyte_Classical_Activation_Inflammatory | *CD14, FCGR1A, S100A8, S100A9, VCAN, CCR2* |
| Monocyte_NonClassical_Patrolling_Activation | *FCGR3A, CX3CR1, CSF1R* |
| **Neutrophil Signatures** |  |
| Neutrophil_Activation_Inflammatory | *FCGR3B, ITGAM, MPO, ELANE, CEACAM8, CXCL8, IL1B, S100A12* |
| Neutrophil_NETosis | *PADI4, MPO, ELANE, CIT* |
| **MDSC Signatures** |  |
| MDSC_General_Suppression_Markers | *ARG1, NOS2, S100A8, S100A9, ITGAM, PTGS2, IDO1, IL4RA* |
| MDSC_M_MDSC_Suppression_Markers | *CD14, FCGR1A, CSF1R, STAT3* |
| MDSC_G_MDSC_Suppression_Markers | *CEACAM8, CXCR2, S100A9, MMP9* |
| **Signaling Pathway Signatures** |  |
| Interferon_Gamma_Response | *IFNG, STAT1, IRF1, CXCL9, CXCL10, CXCL11, IDO1, GBP1, GBP2* |
| Interferon_Alpha_Beta_Response | *IFNA1, IFNB1, STAT1, STAT2, IRF7, IRF9, ISG15, MX1, OAS1, OAS2, IFIT1* |
| Inflammasome_Activation | *NLRP3, PYCARD, CASP1, IL1B, IL18* |
| NFKB_Signaling_Activation | *NFKB1, REL, RELA, NFKBIA, TNF, IL1B, TNFAIP3* |
| TGF_Beta_Signaling_Response | *TGFB1, TGFBR1, TGFBR2, SMAD2, SMAD3, SMAD4, SERPINE1* |
